# Identification of placental nutrient transporters associated with intrauterine growth restriction and pre-eclampsia

**DOI:** 10.1186/s12864-018-4518-z

**Published:** 2018-03-02

**Authors:** Xiao Huang, Pascale Anderle, Lu Hostettler, Marc U. Baumann, Daniel V. Surbek, Edgar C. Ontsouka, Christiane Albrecht

**Affiliations:** 1 Swiss National Centre of Competence in Research, NCCR TransCure, University of Bern, Bern, Switzerland; 2 Institute of Biochemistry and Molecular Medicine, Faculty of Medicine, University of Bern, Bern, Switzerland; 3 Swiss Institute of Bioinformatics and HSeT Foundation, Lausanne, Switzerland; 4 Sitem-insel AG, Bern, Switzerland; 5 Department of Obstetrics and Gynaecology, University Hospital, University of Bern, Bern, Switzerland

**Keywords:** Bioinformatics, Membrane transporters, Placenta, Pre-eclampsia, Intrauterine growth restriction

## Abstract

**Background:**

Gestational disorders such as intrauterine growth restriction (IUGR) and pre-eclampsia (PE) are main causes of poor perinatal outcomes worldwide. Both diseases are related with impaired materno-fetal nutrient transfer, but the crucial transport mechanisms underlying IUGR and PE are not fully elucidated. In this study, we aimed to identify membrane transporters highly associated with transplacental nutrient deficiencies in IUGR/PE.

**Results:**

In silico analyses on the identification of differentially expressed nutrient transporters were conducted using seven eligible microarray datasets (from Gene Expression Omnibus), encompassing control and IUGR/PE placental samples. Thereby 46 out of 434 genes were identified as potentially interesting targets. They are involved in the fetal provision with amino acids, carbohydrates, lipids, vitamins and microelements. Targets of interest were clustered into a substrate-specific interaction network by using Search Tool for the Retrieval of Interacting Genes. The subsequent wet-lab validation was performed using quantitative RT-PCR on placentas from clinically well-characterized IUGR/PE patients (IUGR, *n* = 8; PE, *n* = 5; PE+IUGR, *n* = 10) and controls (term, *n* = 13; preterm, *n* = 7), followed by 2D-hierarchical heatmap generation. Statistical evaluation using Kruskal-Wallis tests was then applied to detect significantly different expression patterns, while scatter plot analysis indicated which transporters were predominantly influenced by IUGR or PE, or equally affected by both diseases. Identified by both methods, three overlapping targets, SLC7A7, SLC38A5 (amino acid transporters), and ABCA1 (cholesterol transporter), were further investigated at the protein level by western blotting. Protein analyses in total placental tissue lysates and membrane fractions isolated from disease and control placentas indicated an altered functional activity of those three nutrient transporters in IUGR/PE.

**Conclusions:**

Combining bioinformatic analysis, molecular biological experiments and mathematical diagramming, this study has demonstrated systematic alterations of nutrient transporter expressions in IUGR/PE. Among 46 initially targeted transporters, three significantly regulated genes were further investigated based on the severity and the disease specificity for IUGR and PE. Confirmed by mRNA and protein expression, the amino acid transporters SLC7A7 and SLC38A5 showed marked differences between controls and IUGR/PE and were regulated by both diseases. In contrast, ABCA1 may play an exclusive role in the development of PE.

**Electronic supplementary material:**

The online version of this article (10.1186/s12864-018-4518-z) contains supplementary material, which is available to authorized users.

## Background

Worldwide, more than one third of child deaths can be attributed to maternal and fetal undernutrition [1]. Along the course of gestation, the fetus receives organic and inorganic nutrients from the mother, such as amino acids, carbohydrates, lipids, vitamins and minerals, for adequate development and growth. To avoid direct materno-fetal blood contact, the placenta has distinctive anatomical barriers for regulating the substrate exchange [2, 3]. Various molecules originating from maternal blood enter the fetal circulation after crossing successively the apical microvillous plasma membrane (MVM), the cytoplasm, and the basal plasma membrane (BM) of the trophoblast layer [3, 4]. On the other side, the fetus eliminates waste products via efflux processes at the BM into the maternal veins. This highlights the crucial role of substrate specific membrane transporter proteins at these different locations. Indeed, for several membrane transporter proteins (e.g. Ca^2+^ -ATPase, ferroportin, system A amino acid transporters) an asymmetric distribution between MVM and BM has been reported. Although this asymmetry is a prerequisite for net transport across the placenta via transporter proteins, the mechanisms of protein trafficking that result in distribution to either MVM or BM in the polarized syncytiotrophoblast are largely unknown. It is evident, however, that any reduction of the quantity, density, and activity of membrane transporters at the placental microvilli could impact the rates of the nutrient supply, which may subsequently compromise the fetal development. It is well recognized that gestational diseases such as intrauterine growth restriction (IUGR) and pre-eclampsia (PE), which complicate a significant proportion of pregnancies leading to poor perinatal outcome, are closely related to impaired placental nutrient transfer [5, 6].

Transfer of amino acids across the placenta is mediated by substrate selective amino acid transport systems. So far, more than 15 different systems have been identified in the human placenta, which include Na^+^-dependent systems A, N, ASC, β, X_AG_−, X_C_−, GLY and Na^+^-independent systems L, y^+^, y^+^L, T, b^o,+^ [5, 7, 8]. Reduced activity of amino acid transporters e.g. system A in IUGR and PE has been reported [9–11], which might be due to reduced sodium-potassium ATPase (Na^+^/K^+^ATPase) activity [12] and a competitive inhibition by physiologically elevated maternal plasma homocysteine [13, 14]. The transplacental transport of glucose mainly occurs along a concentration gradient facilitated by the glucose transporter (GLUT) family, while lipids are mostly packed into circulating protein particles by virtue of active membrane transporters [15]. Current knowledge in this field revealed an increased concentration gradient from maternal to fetal sides in IUGR and PE to achieve higher glucose and lipid transfer [16, 17]. These significant elevations have also been considered as a risk factor for those gestational disorders [18, 19], which may be explained by an altered expression of membrane transporter [20, 21].

The fetal provision with vitamins and dietary elements is required, since they are essential to regulate the fetal metabolism as well as extra- and intra-cellular osmolality. Global studies under the auspices of the World Health Organization in 2008 proposed that deficiencies in micronutrients such as thiamine (Vitamin B1), riboflavin (Vitamin B2), folate (Vitamin B9), cobalamin (Vitamin B12), and ascorbic acid (Vitamin C), are highly prevalent and occur concurrently among pregnant women [1]. It has been reported that the elevated serum homocysteine levels in IUGR and PE were accompanied by decreasing serum levels of the aforementioned class B vitamins. The dietary treatment of pregnant women with diverse vitamin B supplements improved fetal weight [22–26]. However, data regarding the transplacental transport of vitamin B are scarce. In this context SLC19 family members were found to transport folate (SLC19A1) and thiamine (SLC19A2 and SLC19A3) [27]. Interestingly, in IUGR placentas, an increase in mRNA level of SLC19A1 and SLC19A2 has been reported [28], which was interpreted as a compensatory effect. Besides, deficiencies in serum calcium, magnesium and zinc might greatly contribute to the pathogenesis of PE, since it was suggested that the dietary supplementation of these elements can reduce the severity of PE [29–31]. Although calcium is probably the most studied element among all ions, publications investigating the role of transporters or exchangers in placental calcium homeostasis are negligible (< 20). It was found that the expression of Ca^2+^-selective receptor channels and Na^+^/Ca^2+^ exchangers were significantly increased in hypertensive placentae compared to normotensive tissues [32].

**Fig. 1:**
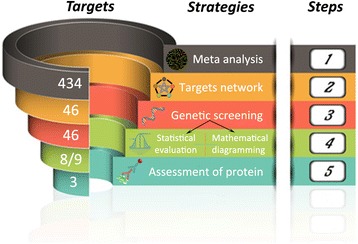
Strategy to target nutrient transporters in intrauterine growth restriction and pre-eclampsia using multiple approaches. The funnel like structure of the different analytical approaches represents the decreasing number of targets which overlap in every step of the analyses. Originating from 434 genes in meta-analysis of microarray data (1), 46 differentially expressed candidate genes were selected and clustered by STRING network (2). Gene expression screening of an in house placental tissue collection in the laboratory (3) was analysed by statistical evaluation and mathematical diagramming (4). The resulting 3 overlapping transporters were finally subjected to the biochemical analyses of protein translation and membrane localization

The studies mentioned above and numerous others have suggested that an altered transplacental nutrient transport might elicit IUGR/PE [5, 6, 33]. However, the detailed underlying mechanisms involving membrane transporters are still not fully elucidated. Therefore, we conducted the current study to gain a comprehensive knowledge on the importance of placental transporter proteins majorly involved in the fetal provision with amino acids, carbohydrates and derivatives, lipids, vitamins, and microelements. We hypothesized that the expression of placental membrane transporters was modified by IUGR/PE. To identify the key nutrient transporters in placenta, we have used a multifaceted approach including bioinformatic analysis, molecular biology, and mathematical diagramming (Fig. 1).

## Methods

### Identification of target transporters by microarray processing

Publicly available data was obtained at http://www.ncbi.nlm.nih.gov/geo through the accession numbers GSE 25861, 24129, 12216, 25906, 14722, 35574, 24818. Robust multi-array averaging (RMA) and quantile normalization were used to quantify gene expression. Significant differences were identified applying a Bayesian approach using the limma package (R 3.1, Bioconductor 2.7). We analysed gene expression data of solute carriers (SLCs), ATP-binding cassette transporters (ABCs) and transient receptor potential channels (TRPs). Any SLC, ABC and TRP family member, which i) was represented in more than 3 studies, ii) was listed in top 12 up- or down-regulated genes, and iii) has an annotated a biological significance in placenta, was added to the candidate list.

### Visualization of biomolecular interaction network

To better understand the network of the identified nutrient transporters from microarray data, we applied the identified targets of interest to Search Tool for the Retrieval of Interacting Genes (STRING) 9.1 at http://string-db.org with a focus on the direct (physical) and indirect (functional) targets’ connections. In the STRING tool, we restricted the prediction to human placenta with medium protein-protein interactions (associated with a confidence score larger than 0.4), and clustered by the Markov cluster algorithm (MCL).

### Placental sample collection

The study was approved by the local ethic institutional review board of the Canton of Bern with an informed consent obtained from each participant prior to the caesarean section. Pregnant women were under obstetrical care at the Department of Obstetrics and Gynecology, University Hospital (Bern, Switzerland). They were assigned to five groups: term controls, preterm, IUGR, PE, and PE combined with IUGR (PE + IUGR). Term controls consisted of the pregnancies without pathologies and terminated at term (≥ 37 gestational weeks) by elective primary caesarean section upon patients’ request or due to breech presentation. The preterm group was defined as the babies born prior to 37 weeks of pregnancy, but without further abnormal maternal or fetal symptoms. According to the definition from the International Society for the Study of Hypertension in Pregnancy, patients with the dramatic increase of systolic/diastolic blood pressure (> 140/90 mmHg) and urinary protein content in 24 h (> 0.30 g) were classified in PE related groups. When the fetal birth weight was below the 10^th^ percentile for babies of the same gestational age, the baby was also referred to as small for gestational age due to the IUGR effects. As it is a severe complication when PE occurs concomitantly with IUGR, we also included and characterized patients suffering from PE combined with IUGR (PE + IUGR).

Placental tissue collection was performed and fully validated as previously described [21, 34]. Immediately after caesarean section, chorionic tissue was dissected from a standardized central location, and snap-frozen in liquid nitrogen after a thorough wash with Dulbecco’s phosphate buffered saline (Gibco). The collected tissue specimens were stored at -80 °C until further analysis.

### Quantitative RT-PCR

Total RNA was isolated from placental tissue with a modified protocol, using a Trizol reagent kit (Invitrogen) [34]. Approx. 50 mg frozen placental tissues were homogenized in 1 ml cold Trizol on ice with a Polytron homogenizer (Kinematica AG), followed by phenol/chloroform extraction and precipitation by isopropanol, and final washing with 75% ethanol. After re-suspension in RNase free water, the extracted RNA was evaluated qualitatively (assessment of RNA integrity) by using Agilent 2100 Bioanalyzer (Agilent Technologies) and quantitatively (determination of RNA concentration) with NanoDrop 1000 (Thermo Scientific). All samples used in this study fulfilled the fixed selection criteria which were: RNA integration number (RIN) > 6, spectrophotometric measurements at 260/280 > 2.0 and 260/230 > 1.8, and the yield of RNA > 5 ng/μl.

Two microgram RNA was reverse transcribed into cDNA using GoScript™ Reverse Transcriptase System (Promega). Quantitative RT-PCR reactions were then performed in 10 μl volume with 1 μl cDNA template, 0.3 μM specific primers (listed in Additional file 1) and GoTaq® qPCR Master Mix (Promega) on the ViiA™ 7 Real-Time PCR System (Applied Biosystems). Based on the results of the transporter mRNA expression, the heatmap was generated using centred correlation (Cluster 3.0) and average linkage clustering analysis for similarity measurement and clustering (TreeView at http://diyhpl.us/~bryan/irc/protocol-online/protocolcache/EisenSoftware.htm). The consecutive statistical analysis and mathematical diagramming served for the identification of significant and disease-specific targets, respectively.

### Total protein and membrane fraction extraction

To extract total protein from placenta, tissue samples were homogenized in ice-cold hypotonic buffer (10 mM Tris-HCl, 10 mM NaCl, 1.5 mM MgCl_2_ and 0.1% Triton X-100, pH 7.4) supplemented with protease inhibitor cocktail (Sigma), and centrifuged at 1000 × g for 10 min at 4 °C. The supernatants were collected in fresh tubes and stored at -80 °C.

Membrane fractions from placenta tissues were extracted using membrane, nuclear and cytoplasmic protein extraction kit according to the manufacturer’s protocol (Bio Basic Inc.). The whole extraction procedure was carried out on ice in the presence of protease inhibitor, phosphatase inhibitor, dithiothreitol and phenylmethylsulfonyl fluoride (PMSF). The “identity” of the extracted cellular membrane fractions was validated by detecting the corresponding expression of the plasma membrane marker Novus Biologicals, the nuclear marker histone H3 (Novus Biologicals) and the cytoskeleton marker β-actin (Sigma).

The protein content of total lysates and membrane fractions was quantified by bicinchoninic acid assay (Pierce), using bovine serum albumin as standard.

### Western blotting

90 μg of the total tissue lysate or 30 μg of membrane fraction was mixed with Laemmli sample buffer and separated by sodium dodecyl sulfate polyacrylamide gel electrophoresis (Bio-Rad) using 10% acrylamide gels. The immobilized bands were then semi-dry transferred to nitrocellulose membranes (GE Healthcare). Blots were blocked with 5% non-fat milk in Tris Buffered Saline with 0.1% Tween-20. The following antibodies were used: rabbit anti-ABCA1 (Novus Biologicals), rabbit anti-SLC7A7 (Novus Biologicals), and rabbit anti-SLC38A5 (Aviva Systems Biology), as well as mouse anti-β-actin or mouse anti-Na^+^/K^+^ ATPase as internal controls. Primary antibodies were first incubated overnight at 4 °C followed by 4 times washing with Tris-buffered saline supplemented with 0.1% Tween 20 (TBST), and then incubation with DyLight 680 or 800 fluorescence conjugated secondary antibodies (Thermo Scientific). The immunoreactive bands were visualized with the Odyssey® Sa Infrared Imaging System (LI-COR) to obtain relative densitometry values.

### Statistical analysis and mathematical diagramming

The comparative threshold cycle (Ct) method was applied to analyze relative gene expression by fold change = 2^−∆(∆Ct)^, where ∆Ct = Ct_target gene_ - Ct_reference gene_ and ∆(∆Ct) = ∆(∆Ct_test_ - ∆Ct_control)_ [35].

Data were statistically evaluated using GraphPad Prism 6 Software. Kruskal-Wallis analysis followed by a Dunn’s multiple comparison test, were used to analyse statistical differences of the clinical records, the mRNA and protein expression data between the term controls and preterm, IUGR, PE and PE + IUGR groups. A *p* value of less than 0.05 was considered statistically significant.

In addition, scatter plotting was applied to estimate the influence of gestational diseases on transporter expression. Each point depicts a nutrient transporter, whose x-axis and y-axis represents the average log 2-fold changes (FC) in the IUGR group and PE group, respectively. To avoid possible bias (individual variation, experimental error, etc.), we selected only those targets, whose expression has been modulated with an absolute FC > 1.14 (i.e. > 114% or < 86%) over the expression in the term controls [36]. Subsequently, the ratio of y to x was considered as a rank variable. When the ratio is close to 0, it means that the predominant effect on transporter mRNA expression is caused by PE. Conversely, when it approaches infinity, the altered transporter is primarily influenced by IUGR. An intermediate state (i.e. a ratio equal to 1 or -1), indicates an equal (1) or an equal but reversed (-1) impact on the transporter expression level by IUGR and PE.

## Results

### Microarray processing

Six placental gene expression studies focusing on IUGR/PE plus one project investigating umbilical cords associated with low birth weight, were included in this meta-analysis (Table 1). These studies represented in total 258 samples, including 129 controls (term and preterm samples), 67 IUGR-related specimens and 62 PE placentas.

Based on the selection criteria for potential transporter candidates described in the section *Methods*, 46 of the 434 genes were chosen for consecutive analysis. These genes encode amino acids, carbohydrates and derivatives, lipids, vitamins, microelements, and other transport channels (Table 2). Among the selected targets, 32 genes exhibited increased expression levels in pathological pregnancies compared to controls, while 14 genes showed the opposite trend.

**Table 1:** Microarray databank information

Citation	Data sources	Publication date	Platform	Sample origin	Sample size			Probes
					Control	IUGR	PE	
Dunk et al. [52]	GSE25861	2012-04-01	Affymetrix	Isolated PIMEC	3 (p)	6^a^		54,675
Nishizawa et al. [53]	GSE24129	2010-09-15	Affymetrix	Placenta	8 (m)	8	8	32,321
Sitras et al. [54]	GSE12216	2008-08-01	Applied Biosystems	Placenta	8 (m)	8^a^		32,878
Tsai et al. [55]	GSE25906	2010-12-10	BeadArray	Placenta	37 (m)		23	48,701
Winn et al. [56]	GSE14722	2009-02-06	Affymetrix	Placenta	11 (p)		12	42,928
Guo et al. [57]	GSE35574	2012-02-07	Illumina	Placenta	40 (m)	27	19	48,701
	GSE24818	2011-10-21	Agilent	Umbilical cord	22	18		37,903

Data used for meta-analysis was obtained by analysing the Gene Expression Omnibus (GEO) at http://www.ncbi.nlm.nih.gov/geo

(p) preterm controls (m) mixture of preterm and term controls

*Abbreviation:* *PIMEC* Fetoplacental microvascular endothelial cell

^a^IUGR is defined as birth weight < 5^th^ percentile

**Table 2:** Selected nutrient transporters according to their regulational signature and physiological significance

Substrate class	Predominant (predicted) substrate	Gene name	Accession number	Protein name	Log (FC)	Representative references
Amino acids	System L, y^+^L, xc-and asc [58, 59]	SLC3A2	NM002394	4F2hc	− 0.42~ 0.28	[60–63]
	Cationic L- amino acids	SLC7A1	NM003045	CAT1	− 0.52~ 0.89	[64–67]
	Large neutral L-amino acids	SLC7A5	NM003486	LAT1	−0.38~ 0.46	[62, 63, 66, 68–71]
	Cationic amino acids, large neutral L-amino acids	SLC7A6	NM003983	y^+^LAT2	−0.23~ 0.39	[69, 72]
		SLC7A7	NM001126105	y^+^LAT1	− 0.44~ 0.89	[72]
	Neutral L – amino acids	SLC7A8	NM012244	LAT2	−0.12~ 0.25	[73]
	Cystine (anionic form), L-glutamate	SLC7A11	NM014331	xCT	−0.10~ 0.53	–
	Gln, Ala, Asn, Cys, His, Ser	SLC38A1	NM030674	SNAT1	−0.28~ 1.24	[68, 74, 75]
	Ala, Asn, Cys, Gln, Gly, His, Met, Pro, Ser	SLC38A2	NM018976	SNAT2	− 0.11~ 0.11	[50, 68, 75–78]
	Gln, Asn, His, Ser	SLC38A5	NM033518	SNAT5	0.01~ 0.21	[49]
	Polyamines, amino acids [79]	SLC12A8	NM024628	CCC9	−0.45~ 1.09	–
Vitamins	Thiamine [27]	SLC19A2	NM006996	THTR1	0.28~ 0.66	[80]
		SLC19A3	NM025243	THTR2	−1.17~ − 0.12	[80, 81]
	Folate	SLC19A1	NM194255	RFC	−0.10~ 0.84	[82–85]
		SLC46A1	NM080669	PCFT	− 0.15~ 0.05	[82, 86]
	Biotin, pantothenic acid [87]	SLC5A6	NM021095	SMVT	0.01~ 0.04	[88, 89]
	Cobalamin [90]	LMBRD1	NM018368	LMBRD1	−0.26~ 0.22	–
	(Pyridoxine) [91]	SLC22A15	NM018420	FLIPT1	−0.14~ 0.10	–
	L-ascorbic acid [92, 93]	SLC23A1	NM005847	SVCT1	0.01~ 0.10	[94, 95]
		SLC23A2	NM005116	SVCT2	−0.38~ 0.43	[95–97]
		SLC23A3	NM144712	SVCT3	− 0.21~ 0.33	–
Microelements and ions	Zn^2+^ [98, 99]	SLC30A1	NM021194	ZNT1	−0.02~ 0.34	[100–102]
		SLC30A2	NM032513	ZNT2	− 0.06~ 0.17	[100, 101, 103]
		SLC30A4	NM013309	ZNT4	−0.21~ 0.05	[100, 101]
		SLC39A1	NM014437	ZIP1	− 0.13~ 0.04	[100]
		SLC39A8	NM022154	ZIP8	0.00~ 0.95	[104]
	Ca^2+^	TRPV6	NM018646	TRPV6	0.02~ 0.43	[32, 105–107]
	e.g. Cl^−^,(COO)_2_^2−^ [108]	SLC4A1	NM000342	AE1	−0.48~ 0.53	[109, 110]
	e.g. SO_4_^2−^ [111]	SLC26A2	NM000112	DTDST	−0.78~ 0.74	[43, 112]
		SLC26A6	NM134426	Pat-1	− 0.09~ 0.47	[112]
	e.g. Na^+^, H^+^ [113, 114]	SLC9A1	NM003047	NHE1	−0.34-0.00	[110, 115–117]
		SLC9B2	NM178833	NHA2	− 0.35~ − 0.34	–
	e.g. I^−^ [111, 118]	SLC5A5	NM000453	NIS	−0.12~ 0.08	[119–122]
		SLC26A4	NM000441	PDS	0.00~ 0.04	[121, 123, 124]
	e.g. Fe^2+^, Zn^2+^ [125]	SLC11A2	NM000617	DMT1	−0.07~ 0.67	[126–128]
Lipids	Cholesterol, phospholipids [129]	ABCA1	NM005502	ABCA1	−0.19~ 0.71	[21, 130, 132]
		ABCG1	NM207629	ABCG1	− 0.20~ 0.33	[130, 131]
	Drugs, toxins [133]	ABCG2	NM004827	ABCG2	−0.70~ 0.48	[134–136]
	e.g. flavonoids [137]	SLC47A1	NM018242	MATE1	−0.03~ 0.33	[138, 139]
	LCFA, VLCFA [140]	SLC27A2	NM003645	FATP2	−0.58~ 1.37	[141, 142]
		SLC27A4	NM005094	FATP4	− 0.04~ 0.05	[142–144]
Carbohydrates (+ metabolic derivatives)	Glucose [145]	SLC2A1	NM006156	GLUT1	0.12~ 0.66	[20, 40, 146]
	Lactate [147]	SLC16A1	NM003051	MCT1	−0.36~ 1.35	[148–150]
		SLC16A3	NM004207	MCT4	− 0.12~ 0.48	[148–151]
		SLC16A4	NM004696	MCT5	− 0.50~ 0.47	[149]
Neurotransmitter	Norepinephrine [152]	SLC6A2	NM001043	NET	−0.31~ 0.54	[153–155]

Forty six entries were ordered alphabetically by gene name; pivotal information of targets including a representative example of accession number, and protein name, substrate (class), and Log (FC) value from meta-analysis is listed

*Abbreviations*: *SLC* solute carrier, *LCFA* long chain fatty acid, *VLCFA* very long chain fatty acid

### Integration of interaction between selected candidates

**Fig. 2:**
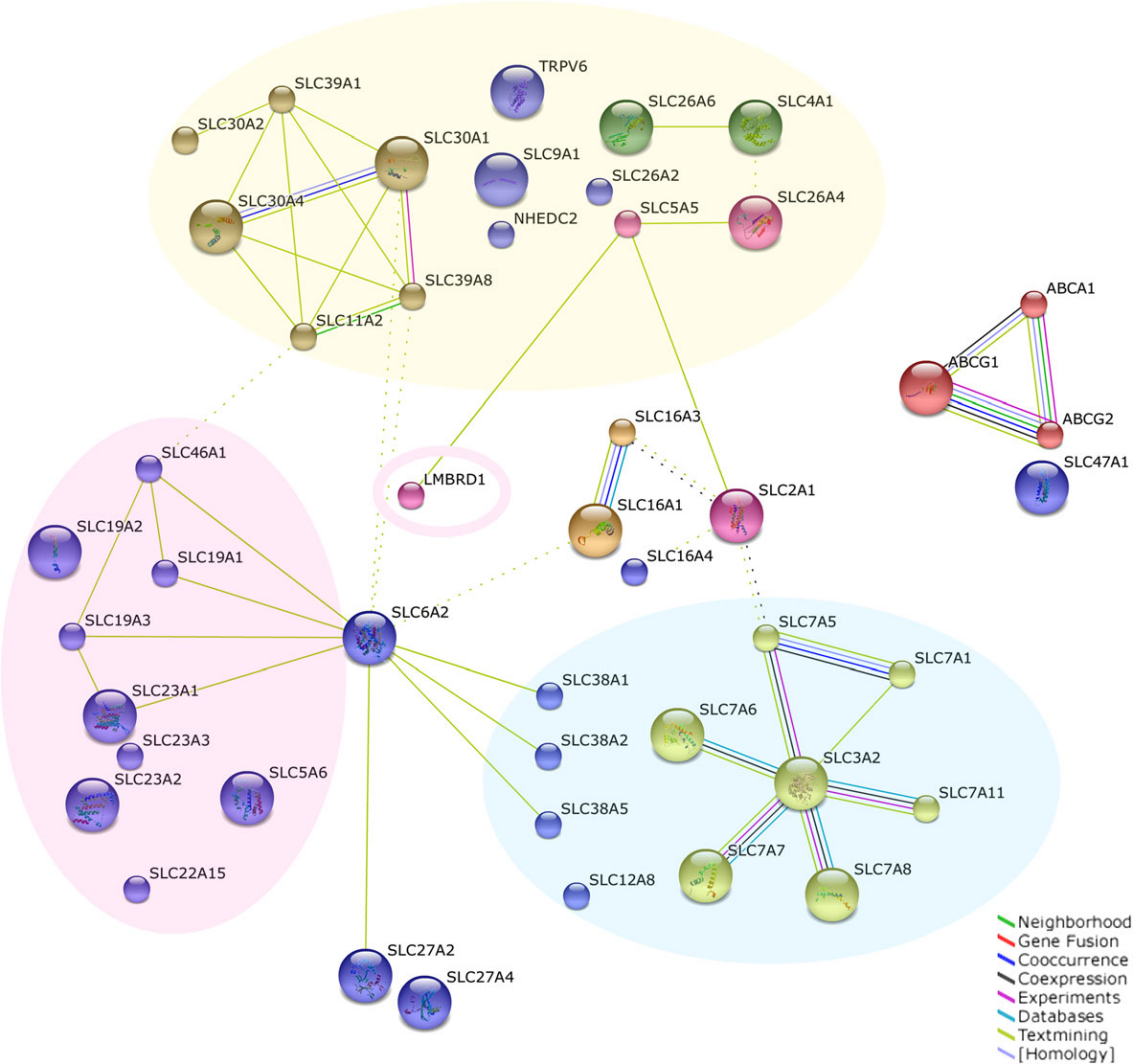
Functional interaction networks of proteins encoded by the potential candidate targets. Forty six transporters selected from microarray analysis were clustered by Search Tool for the Retrieval of Interacting Genes (STRING). The protein-protein interaction in human placenta was performed using customized settings, including medium confidence of 0.4, Markov cluster algorithm (MCL) and 8 criteria for linkage (neighbourhood, gene fusion, co-occurrence, co-expression, experiments, databases, text mining and homology). The red, yellow and blue clusters highlight the substrate-specific components, which are vitamin transporters, microelements and ion transporters, and amino acid transporters, respectively. The available 3D protein structural information is embedded in the node, and the dashed lines represent the inter-cluster edges

As one of the largest database for protein interactions, STRING aggregates the most available information (e.g. neighbourhood, gene fusion, co-occurrence, co-expression, experiments, databases, text mining and homology) derived from genomic context, high throughput experiments, co-expression and previous knowledge. Using the medium confidence score and clustered by MCL, 46 signature nutrient transporters were mapped into an interaction network (Fig. 2). The nodes visualize the proteins with known structure, and the solid lines represent the predicted functional associations with dashed inter-cluster edges. On grouping the network, it summarized the predicted associations for a particular group of proteins, for instance, amino acids associated transporters (blue cluster), vitamins associated transporters (red cluster), microelements and ions associated transporters (yellow cluster). The STRING view depicted that the selected targets were clearly clustered and strongly interconnected based on their substrate specificity, which reflected a high degree of functional association and a potential interplay among the nutrient transporters.

### Identification of altered transporter gene expression in IUGR/PE

#### Characterization of patients

To validate the gene expression on a well characterized, clinically annotated tissue set, we have performed quantitative RT-PCR measurements on a number of accredited representative samples. Table 3 shows the demographic and clinical characteristics of the studied population. There were no major differences in the age, BMI, gravidity and parity numbers between the patients. The gestational age clearly isolated the term pregnancies from the rest, which was directly related with the placental weight and the birth weight.

**Table 3:** Demographics and clinical characteristics of the studied population

	Term	Preterm	IUGR	PE + IUGR	PE	*p*-value
Age, y	33.2	34.0	35.3	32.0	28.7	0.41, ns
	[24.5-41.6]	[29.1-40.7]	[20.9-38.2]	[27.3-40.2]	[23.4-33.3]	
Body mass index (BMI), kg/m^2^	21.3	22.8	24.6	23.3	24.1	0.32, ns
	[18.6-32.8]	[21.5-28.9]	[20.8-35.5]	[19.8-26.0]	[19.8-31.9]	
Gravidity	2.5	2.5	2.0	2.0	1.2	0.09, ns
	[2.0-5.0]	[1.0-6.0]	[1.0-3.0]	[1.0-4.0]	[1.0-2.0]	
Parity	2.0	2.0	2.0	2.0	1.0	0.06, ns
	[1.0-4.0]	[1.0-3.0]	[1.0-3.0]	[1.0-3.0]	[1.0-2.0]	
Gestational age, weeks	38.8	33.8**	35.9*	29.7***	33.7*	< 0.001
	[38.3-39.9]	[26.1-34.6]	[21.7-39.3]	[25.3-36.3]	[25.4-36.0]	
Placental weight, g	547.0	393.5	324.0*	300.0**	365.1	0.003
	[398.0-1035.0]	[250.0-810.0]	[126.0-487.0]	[132.0-481.0]	[203.0-632.0]	
Birth weight, g	3340.0	2127.5*	1850.0**	1170.0***	1973.5 *	< 0.001
	[2950.0-3695.0]	[745.0-2510.0]	[210.0-2695.0]	[520.0-2125.0]	[605.0-2830.0]	
Systolic blood pressure, mmHg	109.5	116.5	126.0	170.0***	164.4**	< 0.001
	[90.0-125.0]	[100.0-146.0]	[109.0-156.0]	[150.0-200.0]	[147.0-213.0]	
Diastolic blood pressure, mmHg	64.0	73.5	75.0	100.0***	98.7***	< 0.001
	[50.0-83.0]	[67.0-91.0]	[63.0-94.0]	[95.0-124.0]	[93.0-104.0]	
Proteinuria, g/24 h	< 0.15	< 0.15	< 0.15	0.88	2.8	
				[0.32-2.54]	[0.5-6.0]	

Statistical analysis was performed using Kruskal**-**Wallis with Dunn’s multiple comparisons test. Data are presented as median with range in brackets; ns, *, **, *** represent a p-value > 0.05, < 0.05, < 0.01, and < 0.001, respectively, of the comparison between the tested group and the term control. Details on patient selection and exclusion criteria are as described in Results (section “Identification of the altered transporter gene expression in IUGR/PE”).

*Abbreviations*: Term, healthy pregnancies at term; Preterm, uncomplicated preterm labour; IUGR, intrauterine growth restriction; PE, pre-eclampsia; PE + IUGR, PE combined with IUGR

In addition, as a PE related biomarker, placental leptin mRNA levels have been measured. A significantly increased expression of leptin has been shown in PE and PE + IUGR compared to both term and preterm control placentae, but not in isolated IUGR (data not shown).

#### Selection of reference genes

Prior to evaluating the nutrient transporter regulation in IUGR/PE, we first screened the mRNA abundance of commonly used reference genes: β2-microglobulin, mitochondrial ribosomal protein L19, ubiquitin (UBQ), and tyrosine 3-monooxygenase/tryptophan 5-monooxygenase activation protein, zeta polypeptide (YWHAZ). We found no differences between controls and the herein tested gestational diseases (data not shown). In accordance with our previous work [34], the mean value of UBQ and YWHAZ was used for quantitative RT-PCR normalization of target nutrient transporters.

#### Gene expression analysis

**Fig. 3:**
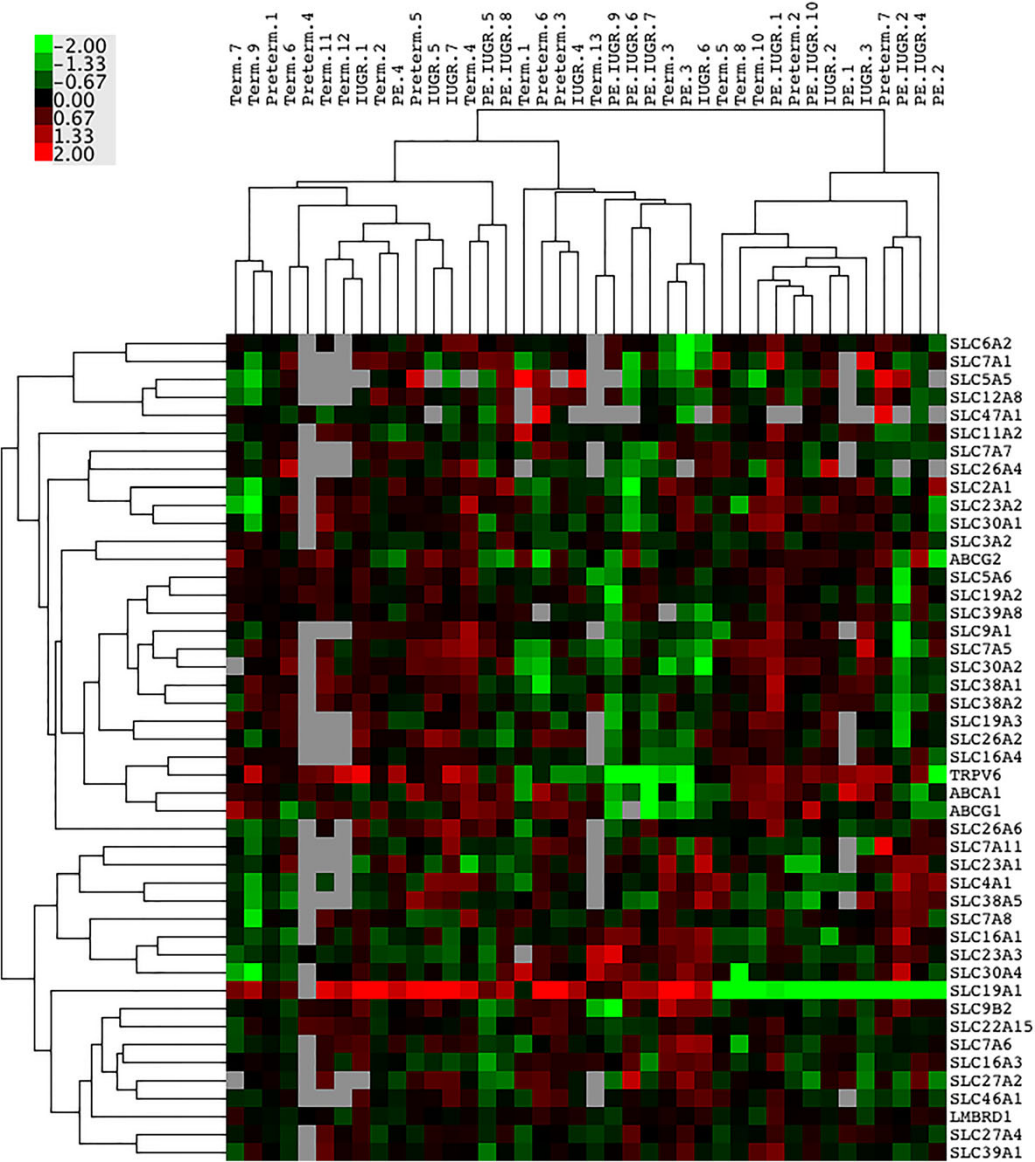
Nutrient transporter mRNA expression profiles of placenta tissues. Heatmap generated from quantitative RT-PCR data from an in-house placental tissue collection, hierarchically clustering the gene expression values of 46 transporters in selected gestational conditions. The clinical status of individual patients, e.g. term (*n* = 13), preterm (*n* = 7), intrauterine growth restriction (IUGR, *n* = 8), pre-eclampsia (PE, *n* = 5), PE combined with IUGR (PE + IUGR, *n* = 10), is indicated on the top, and gene names are listed on the right. Up-regulated expressions are marked in red, down-regulations are coloured in green; black reflects no difference in expression levels

We evaluated the expression of the selected 46 targets in 43 well-characterized placenta samples using unsupervised hierarchical clustering. The heatmap shown in Fig. 3 illustrates an overview of the expression profiles of these transcripts based on the quantitative RT-PCR results. The generated expression profile data confirmed that the 43 well-characterized placenta samples used in this study highly reproduced the results obtained from microarray data of 258 samples all over the world.

**Fig. 4:**
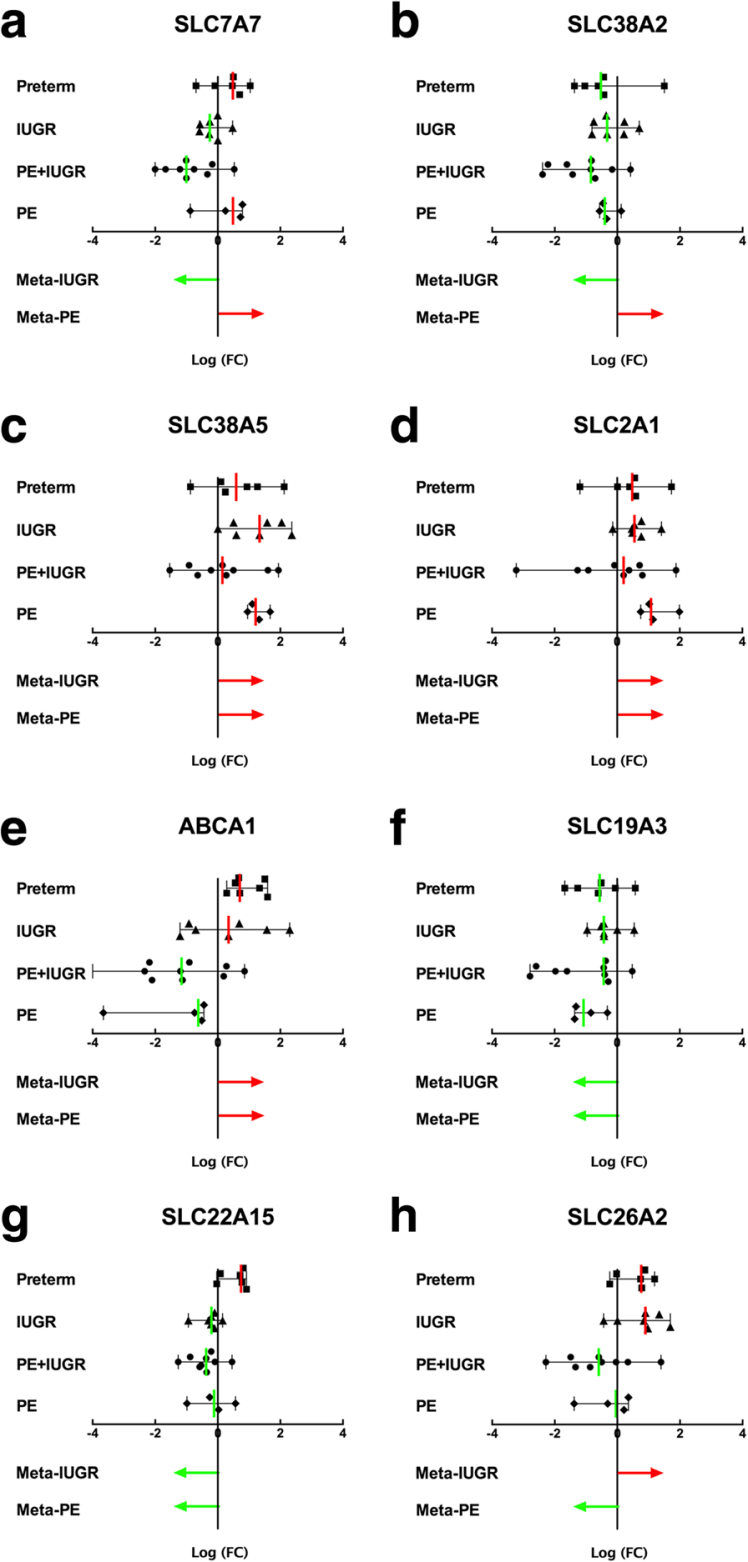
Identification of transporters with a significantly aberrant expression pattern in gestational diseases. The expression patterns of nutrient transporters in placental tissues obtained from controls and the gestational diseases described in Fig. 3 were screened by quantitative RT–PCR. Based on Kruskal**-**Wallis test (*p* <  0.05), eight candidate transporters showed significantly altered mRNA abundance in patients, i.e. SLC7A7 (**a**), SLC38A2 (**b**), SLC38A5 (**c**), SLC2A1 (**d**), ABCA1 (**e**), SLC19A3 (**f**), SLC22A15 (**g**) and SLC26A2 (**h**). Log 2-fold change (FC) values of gene expressions are shown as scatter plots with lines drawn at median ± range. Each symbol represents the gene expression of one individual. In the same figure, the differential expression trends of transporters in IUGR or PE from the meta-analysis (see Fig. 1) are illustrated by arrows. Red and green colours depict up-regulation and down-regulation, respectively

Based on Kruskal-Wallis test, eight candidate transporters were identified showing significant changes on mRNA expression among gestational disorders: the amino acid transporters SLC7A7 (Fig. 4a), SLC38A2 (Fig. 4b), SLC38A5 (Fig. 4c), the glucose transporter SLC2A1 (Fig. 4d), the cholesterol transporter ABCA1 (Fig. 4e), the vitamin B transporters SLC19A3 (Fig. 4f) and SLC22A15 (Fig. 4g), as well as the sulphate transporter SLC26A2 (Fig. 4h). In order to directly compare the trend of gene regulation in IUGR or PE with the microarray analysis, we have presented the gene expression as the log (FC) to term controls. Almost all trends of alteration of transporters by IUGR/PE were comparable to those derived from microarray data, except the effects on ABCA1 and SLC38A2 by PE. The latter might be due to different criteria on PE sample selection, which result in greater variations between microarrays.

**Fig. 5:**
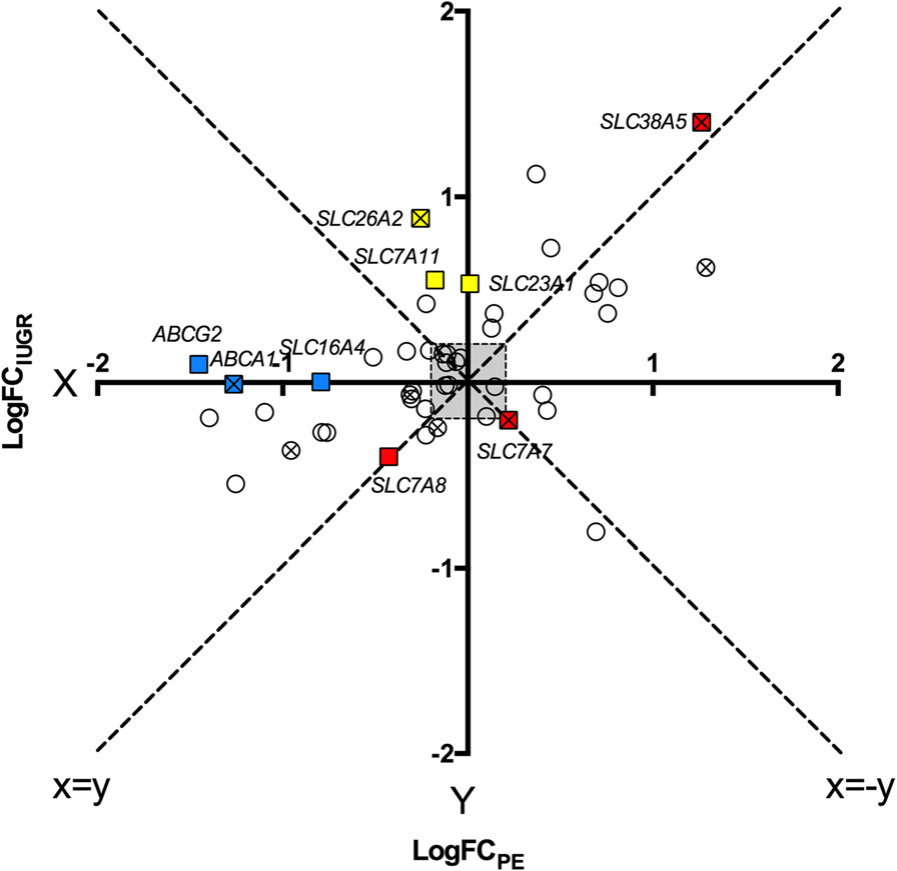
Scatter plot on the influence of IUGR/ PE on transporter expression. Each point represents one nutrient transporter, whose X-axis is defined by its log (FC) expression value in the IUGR group, and the Y-axis is presented by its log (FC) expression value in PE. 0.2 was defined as error threshold. The top 3 genes dominantly influenced by the pathology of IUGR (SLC16A4, ABCA1, ABCG2) or PE (SLC23A1, SLC7A11, SLC26A2), are marked with blue and yellow squares, respectively. Red squares (SLC7A7, SLC7A8, SLC38A5) indicate the transporters that receive comparable effects from IUGR and PE. The points with cross mark depict the significantly regulated transporters by a Kruskal-Wallis test. Abbreviations: log (FC): log 2 fold change

In addition, mathematical diagramming was applied to assess whether IUGR or PE is the major modulator of the determined alteration in gene expression. In the scatter plot shown in Fig. 5, each point represents the average expression level of one nutrient transporter. The log (FC)_IUGR_ and log (FC)_PE_ of independent transporters obtained from quantitative RT-PCR analysis, were defined as paired X-Y values. The absolute value on the X-axis or Y-axis reflects the severity of the gene expression changes by IUGR or PE, respectively. By ranking the absolute X or Y value, the top 3 genes dominantly influenced by IUGR were SLC23A1, SLC26A2, and SLC7A11 (yellow squares), while SLC16A4, ABCA1, and ABCG2 (blue squares) were regulated mainly by PE. On the contrary, transporters such as SLC7A7, SLC38A5 and SLC7A8 (red squares), were equally affected by IUGR and PE since they locate on the y = x or y = −x line.

Taking into account the statistical analysis and the mathematical diagramming, the overlapping targets, namely ABCA1, SLC7A7, SLC38A5, and SLC26A2, were further subjected to protein quantification. However, due to the lack of a specific, validated antibody, the evaluation of SLC26A2 protein could not be further pursued.

### Protein levels of the key membrane transporters

**Fig. 6:**
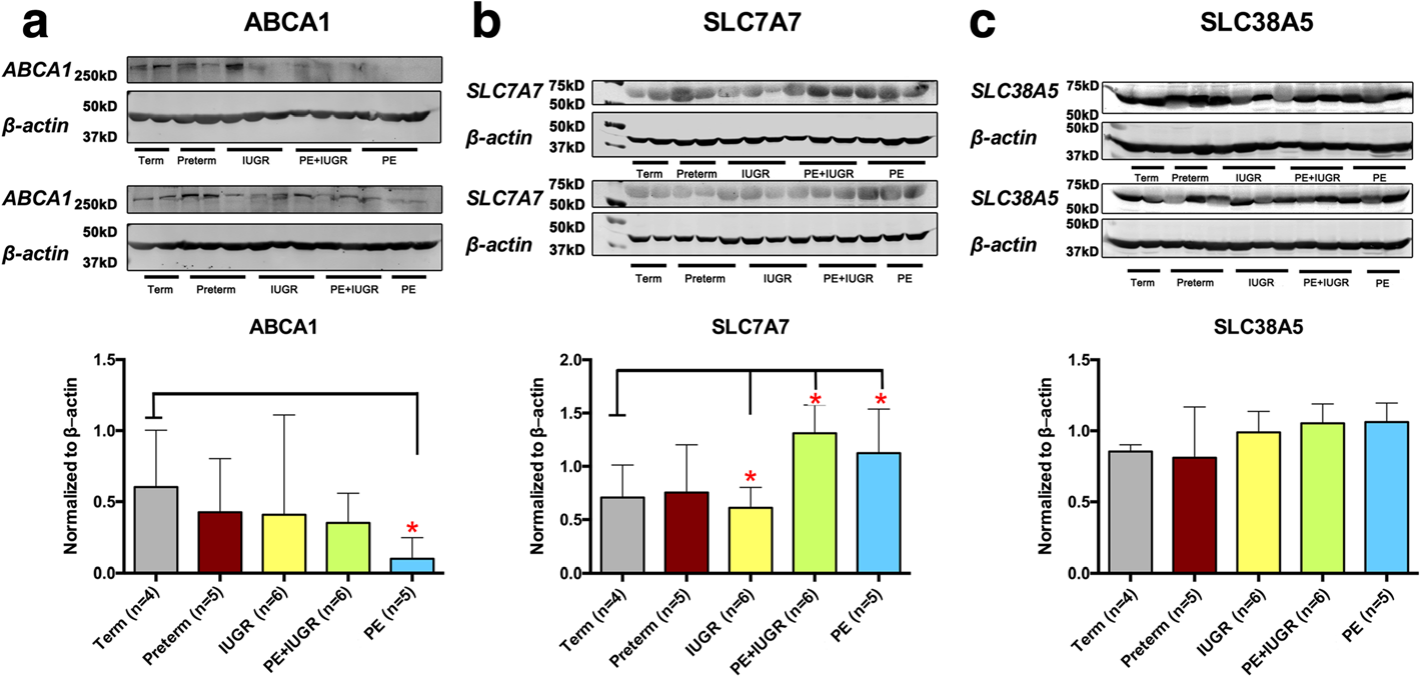
Protein expression of the membrane transporters ABCA1, SLC7A7 and SLC38A5 in total tissue lysates. ABCA1 (**a**; 250 kD), SLC7A7 (**b**; 56 kD), and SLC38A5 (**c**; 51 kD) protein expressions in total lysates were quantified from control and diseased placentas. Western blotting (upper panels) was performed with 90 μg total lysates from term (*n* = 4), preterm (n = 5), IUGR (*n* = 6), PE + IUGR (*n* = 6), and PE (*n* = 5) (upper panels). After overnight exposure, an immunoreactive signal was observed in all lanes at the expected size. Relative protein expression levels from western blotting results were then calculated as the densitometric ratio of target versus β-actin (43 kD; lower panels). Data are expressed as median ± range. Significance at *p* <  0.05 was assessed by a Kruskal-Wallis test, followed by Dunn’s multiple comparison

Using immunoblotting technique, we complemented the quantitative RT-PCR data with corresponding protein expression levels. As shown in Fig. 6, the total protein content of ABCA1 was significantly decreased by PE. In contrast, SLC7A7 transporter showed significant up-regulation in placentae associated with PE, while down-regulation in IUGR placentae was found. SLC38A5 expression in placenta tissue was only slightly elevated in IUGR and PE. The loss of significance may due to the limited sample size. Generally, the changes found on protein level for the three transporters ABCA1, SLC7A7 and SLC38A5, corresponded well with the transcriptional results.

As the target transporters modulate the fluxes of substrates through the plasma membrane to meet fetal requirements, we also isolated plasma membrane protein fractions from the placentae, and confirmed their high purity by enrichment of the marker protein Na^+^/K^+^ ATPase (data not shown). The latter was further used to normalize the transporter protein expression detected by Western blotting.

**Fig. 7:**
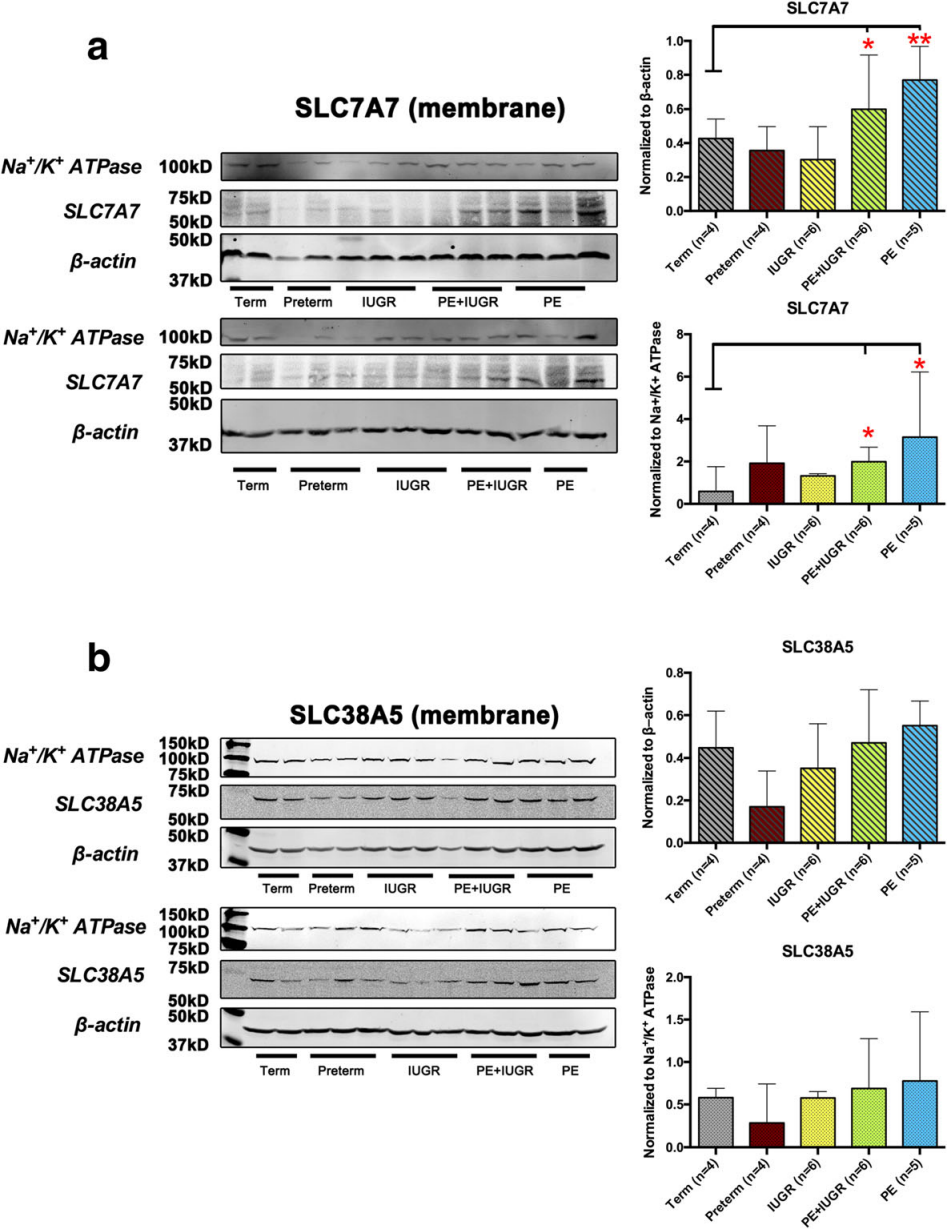
Protein expression of the amino acid transporters SLC7A7 and SLC38A5 in enriched plasma membrane fractions. Representative Western blots of SLC7A7 (**a**; 56 kD) and SLC38A5 (**b**; 51 kD) protein expression from isolated placental membrane fractions. Western blotting was performed with 30 μg membrane proteins from term (*n* = 4), preterm (*n* = 5), IUGR (*n* = 6), PE + IUGR (*n* = 6), and PE (*n* = 5) (Left panels). Both β-actin (43 kD) and Na^+^/K^+^ ATPase (112 kD) were used as loading controls. Relative protein expression levels from western blotting results were then calculated as the densitometric ratio of targeted transporter versus β-actin (43 kD; upper right panel) and Na^+^/K^+^ ATPase (112 kD; lower right panel). Data are expressed as median ± range. Based on Kruskal-Wallis test followed by Dunn’s multiple comparison, *, ** denote *p* < 0.05 and < 0.01, respectively

We have previously reported that the protein expression of the lipid transporter ABCA1 in extracted plasma membranes was significantly reduced only in PE placentae [21]. These findings were confirmed in the present study (data not shown). Interestingly, the opposite regulations for SLC7A7 protein between IUGR and PE-associated disease types that we detected in tissue lysates (Fig.6) were generally also reflected in extracted plasma membrane preparations (Fig. 7). Normalization to β-actin and Na^+^/K^+^ ATPase revealed a trend to decreased expression of SLC7A7 in IUGR tissues, and confirmed a significantly increased expression in PE tissues (Fig. 7a). SLC38A5 was not significantly altered in membrane proteins obtained from diseased and healthy (control) placentas (Fig. 7b). However, marked increases in IUGR and PE compared with the preterm group were found.

## Discussion

Fetal growth is largely dependent on the availability of nutrients transported into the fetal circulation via the placenta. The transport of macro- and micronutrients across the placental barrier relies on the presence and activity of substrate specific membrane transporters. The altered expression of these transporters is thought to be implicated in diverse mechanisms of the fetal development. The current study focused on studying to what extent and which specific transporters are impaired by IUGR or PE. To this end, we screened the expression profiles of nutrient transporters whose substrates are amino acids, carbohydrates, lipids, vitamins and microelements in diseased and control placentas by analysing 7 publicly available GEO microarray datasets. The selected targets have been further validated on in-house well-characterized placenta tissues. A systematic alteration in mRNA expression of diverse transporters were identified in IUGR/PE. Among the 46 nutrient transporters, 8 were significantly regulated by those gestational disorders. In particular, the amino acid transporters SLC7A7 and SLC38A5 were strongly influenced by both IUGR and PE. The mRNA and protein expression of the cholesterol transporter ABCA1, by contrast, was only regulated under PE condition.

Microarray gene expression data provided a global overview on the nutrient transporter expression. However, the weakness of these data was that they were derived from studies using not exactly the same criteria for the clinical recruitment of patients. In this study, we have systematically reviewed our on-site collected samples in the following ways. First, the applied clinical and diagnostic data for each individual did meet the strictly defined clinical criteria for classification as IUGR or PE (Table 3). Second, the higher gene expression of leptin occurring only in the PE and PE + IUGR groups served as a placenta-derived marker of PE [37, 38]. Third, we have compared the regulated mRNA expressions that were generated on our 43 in-house samples with results from the cohort of character-matched 258 patients in seven microarray projects. It showed that only 26% of the targets exhibited inconsistent pathological effects between our samples and the global tissue sets. Based on aforementioned evidences, we considered the in-house tissue samples applied in this study as adequate to reflect patterns occurring in a wider target population.

We further analysed the impact of IUGR/PE on the mRNA expression levels of the selected 46 transporters. Overall, our quantitative RT-PCR measurements revealed a differential expression pattern between control and diseased placentae, including 8 significantly regulated candidates, which might be critical for the fetal malnutrition associated with IUGR/PE. Within this list, SLC2A1 (GLUT1) has been repeatedly reported as significantly reduced at the mRNA level by PE [39, 40]. The studies in animals, such as rat and sheep, have shown that placental GLUT1 expression is significantly decreased in IUGR [41, 42], which is consistent with our findings. We also detected a mRNA down-regulation of the thiamine transporter SLC19A3 and the putative pyridoxine transporter SLC22A15 in IUGR and PE. To our knowledge, this is the first report demonstrating a reduction of placental SLC19A3 and SLC22A15 in IUGR and PE. Currently, there is a lack of specific clinical data on vitamin B1 or B6 deficiency during human pregnancy, except for B vitamin complex supplements. The detected reduction of SLC19A3 and SLC22A15 mRNA in this study may raise our attention to serum thiamine and pyridoxine alterations during pregnancy, which could interrupt the structural and functional development of cerebella and hippocampus, respectively. Information on the sulfate transporter SLC26A2 in placenta is very scarce. Only Simmons et al. recently reported an abundant mRNA and CTB-specific expression of SLC26A2 in human placenta [43]. In the present study, we identified an up-regulation of SLC26A2 mRNA in IUGR and a down-regulation in PE, based on our in silico screening and the in house gene quantitation. Those opposite but significant changes between IUGR and PE may indicate a different role of SLC26A2 in these two diseases which should be further investigated.

The cholesterol transporter ABCA1, as well as the amino acid transporters SLC7A7 and SLC38A5 were consecutively chosen as the main targets for further investigation, as they popped up both in the statistical analysis and the mathematical diagramming procedure. For ABCA1, we have confirmed the previous finding of an exclusively decreased ABCA1 expression in PE [21]. The impaired ABCA1 function resulted in compromised cholesterol and phospholipid efflux, hence a concomitant accumulation of lipids was observed in PE placentas [44]. The amino acid transporter SLC7A7 encodes the y^+^LAT-1 protein, which is known to be located at the MVM and BM [7]. It forms a heterodimer complex with h4F2hc (encoded by SLC3A2), mediating the Na^+^-independent cationic amino acids or large neutral L-amino acids transport [45]. Our finding regarding the decrease of SLC7A7 mRNA and protein in IUGR is in agreement with a previous study using Slc7a7^-^/^-^ mice [46], which caused fetal growth retardation by downregulating insulin-like growth factor 1. In addition, we found an increased SLC7A7 expression in diseases associated with PE compared with gestational age-matched controls. As lysine, arginine and ornithine are substrates for SLC7A7, high SLC7A7 expression could cause reduced maternal lysine, arginine and ornithine levels. During pregnancy this may evoke similar symptoms as reported for lysinuric protein intolerance, which has been associated with serious gestational complications such as increased risk of developing gestational anaemia and PE [47]. SLC38A5, also known as SNAT5 protein, belongs to the Na^+^-coupled neutral amino acid transporter system N. It mediates the cotransport of glycine, asparagine, alanine, serine, glutamine and histidine with sodium [48]. Prior to the current study, SLC38A5 has only been proven to localize at the MVM and to participate in placental glutamine homeostasis [49]. Our work reports for the first time that SLC38A5 is differentially regulated (increased) at mRNA level in the clinically highly relevant pregnancy diseases IUGR and PE. The trend towards an elevated expression as compared to the control groups was also confirmed on protein level, but, supposedly due to the lower sample size and higher variation in the Western blotting experiments, the comparison between the groups in the ANOVA was not statistically different. In comparison, another sodium-coupled neural amino acid transporter, SLC38A2, which is localized at both the MVM and BM [50], was shown to be significantly decreased in IUGR and PE in the mRNA screening (Fig. 4). The differentially altered expression patterns of SLC38A5 and SLC38A2 in IUGR and PE (up- versus down-regulation) might not only result from their differing cellular localizations but also from their distinct transport mechanisms. SLC38A2, as a member of system A, is a pH sensitive uniporter. However, SLC38A5 exhibits marked inhibition at low extracellular pH, because it mediates also the coupled countertransport of H^+^ with the substrate (e.g. glutamine) [51]. Therefore, the uptake or efflux of glutamine via SLC38A5 may strongly depend on the cellular metabolic status, which could be modified in gestational disorders such as IUGR and PE.

## Conclusions

The current study reports a novel analytic strategy including bioinformatic analysis of publicly available microarray datasets, followed by molecular biological and biochemical quantification of mRNA and protein expression in biological tissue samples combined with mathematical diagramming. This approach was successfully applied, firstly to provide an overview on placental membrane transporters providing the fetus with optimal nutrients and microelements for adequate fetal growth, and secondly, to explore the significance of altered membrane transporter expressions in the clinically important pregnancy diseases IUGR and PE. A systematic change in nutrient transporter expression was detected, and 8 membrane transporters were found to be significantly regulated in IUGR/PE. Particularly, the amino acid transporters SLC7A7 and SLC38A5, were influenced by both IUGR and PE. In the present study, we demonstrated for the first time an enhanced mRNA expression of SLC38A5 in IUGR and PE placentae and the same trend on protein level. These findings are the basis for future investigations which should examine function of the selected transporters at different regions in placenta and elucidate the precise mechanisms regulating the observed changes in IUGR and/or PE.

## Additional file

Additional file 1: Gene specific primer sequences used for quantitative RT- PCR. It presents a complete list of the primers designed for the selected candidate and control genes in this study. (DOCX 21 kb)

## Abbreviations

ABC: ATP-binding cassette
BM: Basal plasma membrane
Ct: Cycle threshold
GEO: Gene expression omnibus
GLUT: Glucose transporter
IUGR: Intrauterine growth restriction
log (FC): Log 2 fold change
MCL: Markov cluster algorithm
MVM: Microvillous plasma membrane
Na^+^/K^+^ ATPase: Sodium potassium ATPase
PE + IUGR: PE combined with IUGR
PE: Pre-eclampsia
PIMEC: Fetoplacental microvascular endothelial cell
PMSF: Phenylmethylsulfonyl fluoride
RIN: RNA integrity number
RMA: Robust multi-array averaging
SLC: Solute carrier
STRING: Search tool for the retrieval of interacting genes
TRP: Transient receptor potential
UBQ: Ubiquitin
YWHAZ: Tyrosine 3-monooxygenase/tryptophan 5-monooxygenase activation protein, zeta polypeptide
