# Supplementary material for: Identification of placental nutrient transporters associated with intrauterine growth restriction and pre-eclampsia
Source: BMC Genomics. 2018 Mar 2;19:173. doi: 10.1186/s12864-018-4518-z (PMC5833046; doi:10.1186/s12864-018-4518-z)
Supplement: Supplementary file 1 — Gene specific primer sequences used for quantitative RT- PCR. It presents a complete list of the primers designed for the selected candidate and control genes in this study. (DOCX 21 kb) [file 12864_2018_4518_MOESM1_ESM.docx]

## Additional file 1 – Gene specific primer sequences used for quantitative RT- PCR

| Gene name | Name of gene | Sequence of primers [5`-3`] |
| --- | --- | --- |
| ABCA1 | ATP-binding cassette, sub-family A (ABC1), member 1 | CCACATTTTTGCCTGGGACG  AGCGATTCTCCCCAAACCTT |
| ABCG1 | [ATP-binding cassette, sub-family G (WHITE), member 1](http://www.ncbi.nlm.nih.gov/gene/9619) | AACATGGAGGCCACTGAGAC  GGCCACCAACTCACCACTAT |
| ABCG2 | ATP-binding cassette, sub-family G (WHITE), member 2 | GAGGCAAATCTTCGTTAT  CCATCACAACATCATCTT |
| [LMBRD1](http://www.genecards.org/cgi-bin/carddisp.pl?gene=LMBRD1) | [LMBR1 domain containing 1](http://www.ncbi.nlm.nih.gov/gene/55788) | CCTCTTACTACTGGCTATT  TTATGGTGGAGACAACTT |
| SLC2A1 | solute carrier family 2 (facilitated glucose transporter), member 1 | GAACTCTTCAGCCAGGGTCC  ACCACACAGTTGCTCCACAT |
| SLC3A2 | [solute carrier family 3 (amino acid transporter heavy chain), member 2](http://www.ncbi.nlm.nih.gov/gene/6520) | AGCTGGAGTTTGTCTCAGGC  GGCCAATCTCATCCCCGTAG |
| SLC4A1 | solute carrier family 4 (anion exchanger), member 1 | GATACCTACACCCAGAAACT  GGATGAAGACCAGCAGAG |
| SLC5A5 | solute carrier family 5 (sodium/iodide cotransporter), member 5 | GACCTCATCAAACCTCGGCT  CGTAGATGAGTGAGAGCCCC |
| SLC5A6 | solute carrier family 5 (sodium/multivitamin and iodide cotransporter), member 6 | GTCCTGTACTTTGTGATG  AGGATATAGTGCTGAGAG |
| SLC6A2 | solute carrier family 6 (neurotransmitter transporter), member 2 | GCTCTTGCTCTGTCTGAT  ACACCACCTTTCCTGATG |
| SLC7A1 | [solute carrier family 7 (cationic amino acid transporter, y+ system), member 1](http://www.ncbi.nlm.nih.gov/gene/6541) | CTGGCAGCTCACGGAGGAGGA  GCCCCCGACAGGACACCAGA |
| SLC7A5 | [solute carrier family 7 (amino acid transporter light chain, L system), member 5](http://www.ncbi.nlm.nih.gov/gene/8140) | CAGGGCATCTTCTCCACGAC  TGGGTTCGAGGAGGTGATCTA |
| SLC7A6 | solute carrier family 7 (amino acid transporter light chain, y+L system), member 6 | CCGAGGCAGACAAGTGGAAT  TGTTTGCTGTGGCCTGTCAA |
| SLC7A7 | solute carrier family 7 (amino acid transporter light chain, y+L system), member 7 | AGGCATTTGTCTGGCCTTCCCTTT  TGGCATTGCCTCCTTGGTCCTG |
| SLC7A8 | [solute carrier family 7 (amino acid transporter light chain, L system), member 8](http://www.ncbi.nlm.nih.gov/gene/23428) | AGGCCCTCCTCTGTGGCTGG  GTGCAGCAGGAGTGGCTGGG |
| SLC7A11 | [solute carrier family 7 (anionic amino acid transporter light chain, xc- system), member 11](http://www.ncbi.nlm.nih.gov/gene/23657) | TGCTGGGCTGATTTATCTTCG  GAAAGGGCAACCATGAAGAGG |
| SLC9A1 | solute carrier family 9, subfamily A (NHE1, cation proton antiporter 1), member 1 | GTCATCTTCTTCACCGTCTTTGTG  CGCTTCGTCTCTTGCTTTTTC |
| SLC9B2 | solute carrier family 9, subfamily B (NHA2, cation proton antiporter 2), member 2 | GCCCTGAAGAAGTTAAAG  CCTAAAACAAAACCCAGTA |
| SLC11A2 | [solute carrier family 11 (proton-coupled divalent metal ion transporter), member 2](http://www.ncbi.nlm.nih.gov/gene/4891) | TTGCGGAGCTGGTAAGAATCA  AAGACTGGCAGACTCCCCAT |
| SLC12A8 | solute carrier family 12, member 8 | GGATATTCACCCGAACTG  TACAGAAACCACGAGATG |
| SLC16A1 | [solute carrier family 16 (monocarboxylate transporter), member 1](http://www.ncbi.nlm.nih.gov/gene/6566) | GGGCCACCACTTTTAGGTCGGC  GACGACGCCACATGCCCAGT |
| SLC16A3 | solute carrier family 16 (monocarboxylate transporter), member 3 | GCCCCCTTCGGGAGGCAAAC  GGCCCCCGCCAGGATGAAC |
| SLC16A4 | [solute carrier family 16, member 4](http://www.ncbi.nlm.nih.gov/gene/9122) | TAAGAAGTATCATTACCACAAGTC  ACACAGATCAACCAGTACA |
| SLC19A1 | [solute carrier family 19 (folate transporter), member 1](http://www.ncbi.nlm.nih.gov/gene/6573) | CGAGTCGCAGGCACAGC  CACGGGCACCTGCTTCTC |
| SLC19A2 | solute carrier family 19 (thiamine transporter), member 2 | TCACGATAGCAACTTTTC  GGCATCTACCACAATTAG |
| SLC19A3 | [solute carrier family 19 (thiamine transporter), member 3](http://www.ncbi.nlm.nih.gov/gene/80704) | ACCAGTGCAGAGATAACAA  ACATAATCGGTGAGGACAA |
| SLC22A15 | solute carrier family 22, member 15 | CTGGCTTGTCTTATTGTAA  CAACATTCCTGATGACTG |
| SLC23A1 | solute carrier family 23 (ascorbic acid transporter), member 1 | CCATCAATACAGGCATTCTT  CTTGAGGCTGGAAGACAT |
| SLC23A2 | [solute carrier family 23 (ascorbic acid transporter), member 2](http://www.ncbi.nlm.nih.gov/gene/9962) | TGCAGCCAGCTAGGTCTTGA  AGTGATGTGGCGTAGACCTG |
| SLC23A3 | solute carrier family 23, member 3 | CTGCCAGTTTCATGTGTG  AGACAGTTCCGAAAGGAG |
| SLC26A2 | solute carrier family 26 (anion exchanger), member 2 | GATTGGTGAGACAGTTGAC CCCATCGCTACCTGATAA |
| SLC26A4 | [solute carrier family 26 (anion exchanger), member 4](http://www.ncbi.nlm.nih.gov/gene/5172) | CGTAGATGAGTGAGAGCCCC  GTAGCAATTATCGTCACAAT |
| SLC26A6 | solute carrier family 26 (anion exchanger), member 6 | ACCTACCTGTCAGAACCT  TCCAGCACTGTATAGATGAG |
| SLC27A2 | [solute carrier family 27 (fatty acid transporter), member 2](http://www.ncbi.nlm.nih.gov/gene/11001) | TCTAAGAATACAGGACACCAT  TCAGTCATAGGCACATACAT |
| SLC27A4 | [solute carrier family 27 (fatty acid transporter), member 4](http://www.ncbi.nlm.nih.gov/gene/10999) | CGCTTCGATGGCTACCTCAA  ATCACCAGCACATCACCAGT |
| SLC30A1 | [solute carrier family 30 (zinc transporter), member 1](http://www.ncbi.nlm.nih.gov/gene/7779) | GACCCCGCAGACCCAGAAAA  ACTGAACCCAAGGCATCTCC |
| SLC30A2 | solute carrier family 30 (zinc transporter), member 2 | GAGATGTGATCCTGGTGTT  GTCTGTATTCTGAGCAATGG |
| SLC30A4 | solute carrier family 30 (zinc transporter), member 4 | ACTAGAAGGTGTGCCAAGCC  AGCTGTATGTGAACTATGGCAGT |
| SLC38A1 | [solute carrier family 38, member 1](http://www.ncbi.nlm.nih.gov/gene/81539) | CAGCGCCGGTGCATGTCGA  TGGAAGCTTGACACCCCTGTTAGC |
| SLC38A2 | solute carrier family 38, member 2 | AATGGCTGTGACCCTGACAGTA  TGAGACTATGACGCCACCAACT |
| SLC38A5 | solute carrier family 38, member 5 | AGAGGGTGCCCGAACCTGAGT  TCACGTTCTTGCCTGTAGCCCACA |
| SLC39A1 | solute carrier family 39 (zinc transporter), member 1 | TAGTAAGCTGTTTCGCGGGG  AGTGGGAACTGGAGCGTCA |
| SLC39A8 | [solute carrier family 39 (zinc transporter), member 8](http://www.ncbi.nlm.nih.gov/gene/64116) | GACAGTTATGTTGAGAAGG  TGACCATTCTGACCATAT |
| SLC46A1 | [solute carrier family 46 (folate transporter), member 1](http://www.ncbi.nlm.nih.gov/gene/113235) | ACTCCAAACTAATCGGCTAT  AACCCATATCCTGTGAACA |
| SLC47A1 | [solute carrier family 47 (multidrug and toxin extrusion), member 1](http://www.ncbi.nlm.nih.gov/gene/55244) | CTCAACCAGGGAATTGTA  CGAGGATGTAGAGAAAGA |
| TRPV6 | transient receptor potential cation channel, subfamily V, member 6 | GGTTCCTGCGGGTGGAA  CCTGTGCGTAGCGTTGGAT |
| Leptin | Leptin | CGGATTCTTGTGGCTTTGGC  GAGGAGACTGACTGCGTGTG |
| β2MG | β2-microglobulin | GATGAGTATGCCTGCCGTGTG  CAATCCAAATGCGGCATCT |
| L19 | mitochondrial ribosomal protein L19 | CCAACTCCCGTCAGCAGATC  CAAGGTGTTTTTCCGGCATC |
| UBQ | ubiquitin C | TCGCAGCCGGGATTTG  GCATTGTCAAGTGACGATCACA |
| YWHAZ | tyrosine 3-monooxygenase/tryptophan 5-monooxygenase activation protein, zeta polypeptide | CCGTTACTTGGCTGAGGTTG  AGTTAAGGGCCAGACCCAGT |
